# Twelve Ways of Injuring the Health

**Published:** 1886-09

**Authors:** 


### *TWELVE WAYS OF INJURING THE HEALH.*

1. Wearing of thin shoes and stockings on damp nights and in cool, rainy weather. Wearing insufficient clothing, especially upon the limbs and extremities.

2. Leading a life of unfeeling, stupid laziness, and keeping the mind in an unnatural state of excitement, by reading trashy novels. Going to the theatres, parties and balls, in all sorts of weather in the thinnest dress; dancing till in a complete perspiration, and then going home without sufficient overgarments, through the cool, damp night air.

Sleeping on feather beds in 7 by 9 bed-rooms, without ventilation at the top of the window; especially with two or more persons in the same small unventilated bed-room.

4. Surfeiting on hot and very stimulating dinners; eating in a hurry, without half masticating the food, and eating heartily before going to bed, when the mind and body are exhausted by the toils of the day and the excitement of the evening.

5. Beginning in childhood on strong tea and coffee, and going from one step to another, through chewing and smoking tobacco and drinking intoxicating liquors, and personal abuse, and mental and physical excesses of other kinds

6. Marrying in haste and getting an uncongenial companion, and living the remainder of life in mental dissatisfaction, cultivating jealousies and domestic broils, and being always in a mental ferment.

7. Keeping children quiet by giving paregoric and cordials, by teaching them to suck candy, and by supplying them with raisins, nuts and rich cakes; when they are sick by giving them mercury, tartar emetic and arsenic, under the mistaken notion that they are medicines and not irritant poisons.

8. Allowing the love of gain to absorb our minds, so as to leave no time to attend to our health; following an unhealty occupation, because money can be made by it.

9. Tempting the appetite with bitters and niceties when the stomach says no, and by forcing food into it when nature does not demand, and even rejects it; gormandizing between meals.

10. Contriving to keep a continual worry about something or nothing; giving away to fits of anger,

11. Being irregular in all habits of sleeping; and eating too much, too many kinds of food, and that which is too highly seasoned.

12. Neglecting to take proper care of ourselves, and not applying early for medical advice when disease first appears, but by taking "celebrated" quack medicines to a degree of making a drug shop of the body.—*Appalachian Philosopher.*

